# Maximizing Survival in Pediatric Congenital Cardiac Surgery Using Machine Learning, Explainability, and Simulation Techniques

**DOI:** 10.3390/jcm13226872

**Published:** 2024-11-15

**Authors:** David Mauricio, Jorge Cárdenas-Grandez, Giuliana Vanessa Uribe Godoy, Mirko Jerber Rodríguez Mallma, Nelson Maculan, Pedro Mascaro

**Affiliations:** 1 Department of Computer Science, Universidad Nacional Mayor de San Marcos, Lima 15081, Peru; dmauricios@unmsm.edu.pe (D.M.);; 2 Instituto Nacional de Salud del Niño, Breña, Lima 15083, Peru; guribeg@insn.gob.pe; 3 Facultad de Ingeniería Industrial y de Sistemas, Universidad Nacional de Ingeniería, Lima 15333, Peru; 4 Systems Engineering-Computer Science and Applied Mathematics, CT & CCMN, Campus: Ilha do Fundão, Federal University of Rio de Janeiro, Rio de Janeiro 21941-617, Brazil; maculan@cos.ufrj.br; 5 Faculty of Medicine, Universidad Nacional Mayor de San Marcos, Lima 15081, Peru

**Keywords:** pediatric and congenital heart surgery, prognosis, machine learning, explainability, simulation, intelligent system

## Abstract

**Background:** Pediatric and congenital heart surgery (PCHS) is highly risky. Complications associated with this surgical procedure are mainly caused by the severity of the disease or the unnecessary, late, or premature execution of the procedure, which can be fatal. In this context, prognostic models are crucial to reduce the uncertainty of the decision to perform surgery; however, these models alone are insufficient to maximize the probability of success or to reverse a future scenario of patient death. **Method**: A new approach is proposed to reverse the prognosis of death in PCHS through the use of (1) machine learning (ML) models to predict the outcome of surgery; (2) an explainability technique (ET) to determine the impact of main risk factors; and (3) a simulation method to design health scenarios that potentially reverse a negative prognosis. **Results**: Accuracy levels of 96% in the prediction of mortality and survival were achieved using a dataset of 565 patients undergoing PCHS and assessing 10 risk factors. Three case studies confirmed that the ET known as LIME provides explanations that are consistent with the observed results, and the simulation of one real case managed to reverse the initial prognosis of death to one of survival. **Conclusions**: An innovative method that integrates ML models, ETs, and Simulation has been developed to reverse the prognosis of death in patients undergoing PCHS. The experimental results validate the relevance of this approach in medical decision-making, demonstrating its ability to reverse negative prognoses and provide a solid basis for more informed and personalized medical decisions.

## 1. Introduction

Congenital anomalies are conditions that develop before birth and, in many cases, are detected in the first days of the newborn’s life [1]. These defects in the heart’s structure can alter normal blood flow [2] and, depending on the severity, may require surgical intervention immediately after birth [3]. According to estimates by the United Nations Children’s Fund (UNICEF) in 2023, congenital anomalies were the sixth-leading cause of death in children under five years old worldwide, representing 8% of the total [4]. Among these, congenital heart disease is one of the most frequent.

In general, cardiac surgeries in children are high-risk. On the one hand, pediatric patients may suffer serious complications, such as injuries during surgery that, in some cases, can lead to death. On the other hand, cardiac surgeons face the challenge of making crucial decisions, for example, deciding the right time to perform the surgery, since performing it too early or too late can worsen the results. Therefore, decision-making in pediatric cardiac surgeries is a complex process that involves evaluating multiple risk factors such as weight, age, sex, comorbidities, and preoperative and intraoperative conditions. These factors can significantly influence the outcome, making it difficult to predict whether surgery guarantees survival or results in serious complications. For example, an analysis of 140 cardiac malpractice claims from the VerdictSearch database (http://verdictsearch.com, accessed on 4 May 2023) found that cardiac surgeons were charged in 47.8% of cases and cardiologists in 56.4%. Of these cases, 11.9% involved congenital heart disease, with the most common cause of claim (37.5%) being perioperative injuries related to perfusion problems [5].

Most studies predicting mortality in pediatric and congenital heart surgery (PCHS) use the EuroSCORE II model, which is based on the statistical method named Logistic Regression (LR) that identifies the relationship between risk factors (independent variables) and the outcome of surgery (dependent variable). Another widely used method is the Risk Adjustment for Congenital Heart Surgery (RACHS-1), published in 2002 [6]. However, in recent years, machine learning (ML) algorithms have shown better performance in predicting mortality than traditional methods such as EuroSCORE II; for example, the study conducted by Allyn et al. [7] showed a lower accuracy of conventional methods in predicting mortality in patients undergoing heart surgery. This would be because ML algorithms can build more robust predictive models by analyzing large data sets and finding complex relationships between risk factors and surgical outcomes [8]. However, although ML-based studies could offer highly accurate predictions, surgeons often do not fully trust these results, as the reasoning behind the algorithms could be challenging to understand and interpret. Furthermore, it is not always possible to identify the risk factors that significantly influence the outcome prognosis.

Therefore, since there are currently no studies exploring the possibility of altering or simulating the adverse prognosis of PCHS to maximize the probability of survival, this work seeks to answer the question: *Is it possible to change the mortality prediction outcome in pediatric patients undergoing PCHS?* To this end, we propose an intelligent system that is capable of predicting mortality, identifying the most influential risk factors, and simulating scenarios in which the physician could modify the risk factors to maximize the probability of success of the surgery and, therefore, change the possible adverse outcome of patients undergoing PCHS.

The main contributions of this article are as follows:

- Propose a new method to evaluate the possibility of modifying the clinical prognosis of mortality in PCHS using ETs in predictive models based on ML and simulation scenarios;
- Provide a systematic process for predicting mortality in patients undergoing PCHS, identify the main risk factors that explain the current outcome, and simulate scenarios that could change the patient’s adverse outcome.

This paper is organized into five sections: Section 2 reviews ML-based predictive algorithms and their application in mortality prediction in cardiac surgeries. Section 3 describes the proposed method to modify the mortality prognosis of patients undergoing PCHS. Section 4 presents the validation of the proposed method through numerical experiments and case studies. Finally, Section 5 and Section 6 present discussions and conclusions.

## 2. Background

### 2.1. Machine Learning (ML)

ML refers to developing algorithms and techniques that are capable of learning and performing tasks from large volumes of data without being explicitly programmed for it. These models are simplified representations of reality based on historical data [9]. ML algorithms fall into two main categories: supervised and unsupervised. Both algorithms look for patterns in the data; while supervised ones require labeled data, unsupervised ones do not need labels [10].

Several ML algorithms have been applied in predicting outcomes in cardiac surgery, which are briefly described below:

*Multilayer Perceptron (MLP)* is a technique that simulates a natural neural network and is composed of three or more layers: an input layer, intermediate layers (also called hidden layers), and an output layer (also called results). Its learning is supervised, and the most commonly used training algorithm is called Backpropagation, which adjusts the weights of the neural connections iteratively [11].

*Self-Organizing Map (SOM)* is an unsupervised algorithm developed by Teuvo Kohonen. It is characterized by applying dimensional reduction to find patterns in data. Its architecture consists of two layers: an input layer, where the data are processed, and another that adjusts the weights and agglomerates the found patterns (clusters) [12].

*Radial Basis Function Network (RBF)* is a type of supervised Artificial Neural Network (ANN), similar to an MLP network, but it uses a radial basis function in its hidden layer. RBF converts the input to a higher dimension and performs the classification using only one layer of neurons with linear activation functions [13].

*Iterative Dichotomiser 3 (ID3)* is a supervised algorithm that uses a top-down greedy approach to build several decision trees. It splits iteratively by selecting the best categorical feature to create a node. In its splitting process, it uses the Information Gain (IG) metric to choose the most important features [14].

*C4.5* is a decision tree classifier made up of nodes and leaves. Each node splits the classes concerning the information ratio, i.e., more information is used as a splitting rule. In addition, it presents a “pruning” function that eliminates branches that do not contribute to the decision process, thus reducing the size of the tree and decreasing the error rate [15].

*Gradient Boosting Machine (GBM)* is an algorithm that combines decision trees through a sequential process (Boosting). The algorithm builds trees sequentially so that each new tree corrects the errors of the previously trained tree, creating an increasingly robust predictor [16].

*Extreme Gradient Boosting Machine (XGBoost)* is a supervised algorithm, a variant of GBM, that introduces improvements in regularization, parallel processing, and execution speed and improves performance [17].

*Random Forest (RF)* is an algorithm that combines multiple decision trees. This algorithm trains each tree independently using random samples of data, which reduces the risk of overfitting and improves generalization [18].

*Support Vector Machine (SVM)* is a supervised algorithm that searches for a hyperplane in a multidimensional space that separates the classes to be predicted. It uses kernel functions to transform the data to a higher dimension, facilitating learning. Some kernels are the polynomial, the radial basis, and the sigmoid [19].

*Naive Bayes (NB)* is a simple and fast supervised algorithm whose operation is based on Bayes’ theorem. It assumes that the data features are independent, simplifying its implementation [20].

*Recurrent neural network (RNN)* is a type of ANN that does not have a defined layer structure and allows the analysis of time series data to deal with the “time” dimension [21]. The structure of an RNN allows the results of a layer to feed back to themselves or to feed back to previous layers. This allows it to analyze time series and capture dependencies over time, which is critical to dealing with the temporal dimension in the data.

Finally, the usage of the *K-Nearest Neighbor (KNN)* technique has also been identified. This technique classifies new data by finding the closest points in the learned data space. The value of “k” indicates the number of nearest neighbors to consider, and the classification is performed by majority voting [22].

### 2.2. ML in Cardiac Surgery Prognosis

Several studies have explored the use of ML models for mortality prediction in a healthcare setting. These studies include elderly patients [23], premature infants [24], patients with a high risk of postoperative complications and mortality [25,26,27], adult cardiac arrest cases [28], pediatric patients [29], patients with cardiovascular diseases [30,31,32,33], and patients undergoing heart transplantation [34,35].

A literature review on cardiac surgery outcome prediction performed with ML models in databases such as Scopus, Web of Science (WoS), and PubMed, using the search string “((heart OR cardiac) AND surgery) AND (mortalit* OR morbid*) AND (prediction OR forecast*)” in titles, abstracts, and keywords”, showed a limited number of related studies (see Table 1). From the search results, only two studies were directly related to PCHS [36,37]. While the study by Jalali et al. [38] focuses on hypoplastic left heart syndrome for PCHS, the remaining ones are oriented to standard cardiac surgery. Furthermore, the ML algorithms used in these studies are varied, and no standard validation dataset has been identified. Finally, the reported performance metrics are diverse, with values for accuracy varying in the range of 61% and 99%, and values for Area Under the Curve (AUC) ranging between 80% and 88%.

## 3. Materials and Methods

**Figure 1:**
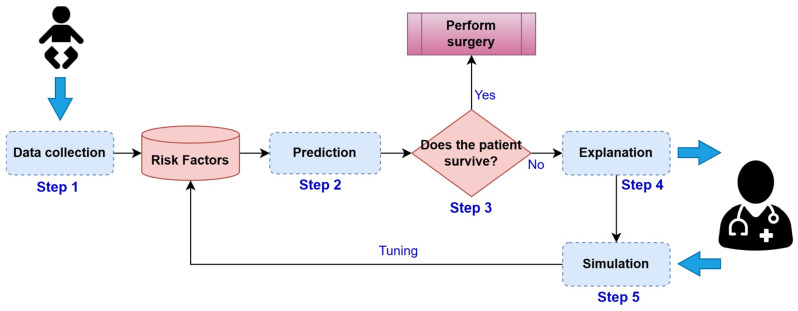
Method for changing the mortality outcomes of patients undergoing PCHS.

A method is proposed that integrates ML algorithms, ETs, and simulation scenarios to maximize survival predictions in PCHS, as illustrated in Figure 1.

### 3.1. Proposed Method

The method consists of five steps, following the logic in Figure 1. Step 1 (capture) collects the patient’s risk factor data. In Step 2 (prognosis), PCHS mortality is predicted using ML. Step 3 (evaluation) evaluates whether the prognosis anticipates a case of survival, then the method concludes, and the surgery is carried out; otherwise, it continues with the next step. In Step 4 (explanation), the influence of each risk factor on the prognosis is determined using ETs, and it is sent to the medical specialist for analysis and evaluation. In Step 5 (simulation), based on the previous analysis of Step 4, the specialist adjusts the risk factor values (if possible), and, with these new values, returns to Step 2. Steps 2–5 are repeated as many times as necessary until the prognosis is changed from mortality to survival. If this is achieved, the probability of changing the future from death to survival in PCHS will have been maximized.

To build the ML models, ETs, and simulation scenarios, the programming language Python version 3.7.5 and the library scikit-learn version 1.0.2 were used within the Google Colab development environment.

#### 3.1.1. Step 1—Data Collection

In this step, the pediatric patient’s personal, clinical, and demographic data are collected, taking into account the main risk factors for PCHS identified in the literature, which are classified as preoperative and intraoperative. Preoperative factors include age in days from birth up to 5 years [38,39,42,43], sex [7,38,39,40,41,42,43], weight in kilograms [38,39,43], prematurity (less than 36 weeks of gestation) [43], and preoperative serum creatinine levels (ranging from 0 to 1.3) [43]. Intraoperative factors (those that manifest during surgery) include antifibrinolytic drugs such as aprotinin and tranexamic acid [38,43], cardiopulmonary bypass time in minutes [38,39,43], heart pump [43], deep hypothermic circulatory arrest time in minutes [38,43], and aortic cross-clamp time in minutes [38,39,43]. Thus, ten risk factors were identified (five preoperative and five intraoperative) and used in the present study.

#### 3.1.2. Step 2—Prediction

This step predicts PCHS mortality using an ML model and historical risk factor data previously pre-processed. Pre-processing includes qualitative to quantitative data transformation, class categorization, and data normalization to ensure uniform scaling. The ML model is obtained following standard processes similar to those used in studies such as Bruise dating [44] and Keratoconus detection [45]. The algorithms used to build the models include XGBoost [39], RF [38], DT [38], NB [7], SVM [7], KNN [40], and RNN [41].

#### 3.1.3. Step 3—Evaluation

In this step, the prediction’s outcome is evaluated. If a high probability of survival is predicted, the process is concluded, and the PCHS is performed. Otherwise (i.e., the prediction indicates a mortality risk), the method advances to Step 4 to try to reverse this prediction.

#### 3.1.4. Step 4—Explanation

In this step, the degree of influence of each risk factor on the mortality prognosis from Step 2 is determined. These degrees of influence are crucial to understanding how various factors contribute, positively or negatively, and the different levels of impact (high, medium, low) to the patient’s mortality outcome. For this purpose, an ET is used. The ETs are essential tools to implement what is also known as interpretability in the artificial intelligence (AI) field [46]. These techniques allow us to explain how and to what extent the factors positively or negatively affect the final result of the prognosis. Although they are used in conjunction with the predictive models, they are independent of them. Some ET algorithms are Anchors [47], SHAP [48], LORE [49], LIME [50], and MAPLE [51].

#### 3.1.5. Step 5—Simulation

In this step, the degree of influence of the risk factors obtained in Step 4 is analyzed and evaluated, and the values of these factors are modified to mitigate the adverse effects and/or enhance the positive ones in such a way as to increase the probability of PCHS survival. This adjustment is made by the specialist surgeon according to their experience, within what is permissible and based on the rules generated by the ET; that is, the patient is simulated with values of risk factors modified to maximize the probability of postoperative survival.

In a scenario where changing the prediction from mortality to survival is impossible, it is recommended to follow the standard medical protocol and use the model’s prediction as a more accurate medical decision-support input.

### 3.2. Dataset

This study used the dataset of pediatric patients undergoing cardiac surgery, extracted from the article “The Safety and Efficacy of Antifibrinolytic Therapy in Neonatal Cardiac Surgery” [43], for the training, validation, and testing of the models of the proposed method. Ten preoperative and intraoperative variables identified in the literature (see Step 1) were used. The categorical variables were coded, the values of the age factor were transformed from text to numeric, and the target categories of “survival” and “die” were renamed to 0 and 1, respectively. The number 1 indicates the death of the patient and 0 indicates the survival. In addition, the dataset was balanced to have the same number of records in both the “die” and “survival” categories, using the “resample” method in the scikit-learn library [52]. The number of records in the preprocessed dataset was increased by 385, from the original 565 records to 950. Of the preprocessed dataset, 90% (855 records) were used for training and validation, and the remaining 10% (95 records) for testing (see Table 2).

### 3.3. Cross-Validation

To prevent overfitting and ensure a good generalization of the ML model, cross-validation with k = 10 was implemented. Models were trained and validated using 90% of the processed dataset. The training and validation dataset was divided into 10 equal parts; in each iteration, 9 were used for training and 1 for evaluation. Each iteration was repeated 10 times to ensure that each part was used in the evaluation. The random search method called RandomizedSearchCV [53] was used to obtain the optimal hyperparameters for the ML algorithms. The final parameters are presented in Table 3.

### 3.4. Metrics

Accuracy, sensitivity, and specificity metrics [41] were used to evaluate the models’ performance. Table 4 details the definitions of these metrics.

## 4. Results

This section shows the results of the experiments with the implemented forecasting model, the simulation, and the rules obtained from explainability.

### 4.1. Prediction Results

Four ML models (GBM, RF, DT, and BN) were evaluated using a Lenovo computer equipped with an Intel Core i5 processor, sourced from Lima, Peru, with 8 GB of RAM, 617 GB of available disk space, and Windows 10 Home 64-bit operating system.

First, the scikit-learn library was used to calibrate each model’s hyperparameters, obtaining the results in Table 3. Next, training and cross-validation were performed with the Training & Validation dataset (see Table 2), spending approximately 10 min for each model. Finally, testing was performed using the Test dataset (see Table 2), obtaining the confusion matrix in Table 5.

From the confusion matrix, the accuracy, sensitivity, and specificity metrics were calculated for the four ML models (see Table 6). The GBM and RF models obtained the best performances in terms of accuracy, with values of 95% and 94%, respectively, as well as a sensitivity of 100% and specificity greater than 90%. These results exceed those previously reported in the literature [39].

### 4.2. Explainability Results

The GBM model was selected as the prediction model due to its superior results compared to the other evaluated models, and the ET known as LIME was used for explainability purposes.

The explainability results include three components: the prognosis, the score, and the bar graph. The prognosis can be mortality or survival. The score indicates the probability associated with each outcome, where a score of 1 indicates 100% certainty in the occurrence of that specific outcome. A score of “s” for mortality is equivalent to “(1 − s)” for survival; for example, a mortality score of 0.15 (15%) implies an 85% probability of survival. Also, the bar graph illustrates how each of the 10 PCHS risk factors influences the outcome. The size of each bar reflects the level of influence, while the bar’s color indicates its level of impact: green (left) for survival and red (right) for mortality.

### 4.3. Case Studies

Below are the results of three case studies in which three forecast scenarios were analyzed: correct mortality, correct survival, and incorrect mortality.

#### 4.3.1. Correct Mortality Prediction

The actual case of a 6-day-old male pediatric patient diagnosed with congenital heart malformation, weighing 1.4 kg, premature, and undergoing cardiopulmonary bypass surgery for 157.0 min was analyzed. Aprotinin was administered as an antifibrinolytic drug, while 14 min of deep hypothermic circulatory arrest and 67 min of aortic cross-clamping were recorded, with a preoperative serum creatinine of 0.9. According to records, the patient died after surgery.

**Figure 2:**
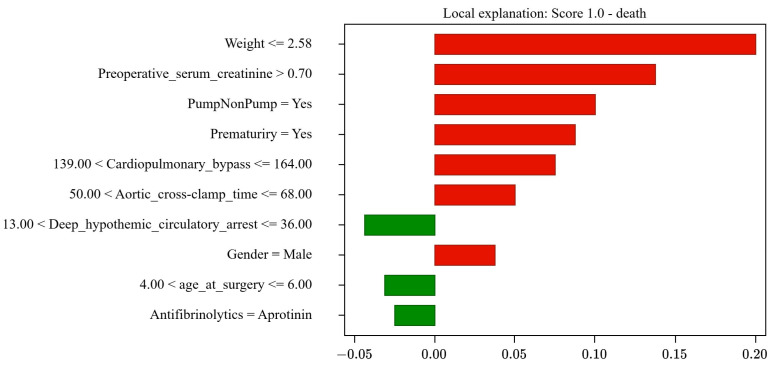
Results of Case Study 1: Correct mortality prediction (100%).

The system correctly predicted the patient’s mortality, assigning a survival probability of 0%, as shown in Figure 2. Table 7 identifies the factors that influenced mortality: low weight (less than 2580 kg) with a 21% influence, creatinine greater than 0.7 (13%), the use of cardiac pumping (9%), a history of prematurity (8%), long cardiopulmonary bypass time (7%), prolonged aortic clamping time (4%), and male sex (3%). The positive factors identified were the reduced deep hypothermic circulatory arrest time of 14 min (4%), the use of antifibrinolytics (3%), and the patient’s age (3%).

More factors with negative influence (red bars) than positive ones (green bars) explain the high probability of mortality.

#### 4.3.2. Correct Survival Prediction

The actual case of a 28-day-old male pediatric patient diagnosed with congenital heart malformation, weighing 3.65 kg, not premature, and undergoing cardiopulmonary bypass surgery lasting 67.0 min was analyzed. No antifibrinolytic was used, and the deep hypothermic circulatory arrest time was 0.0 min, with an aortic cross-clamp time of 35.0 min. Preoperative serum creatinine was 0.3. According to records, the patient survived the operation.

**Figure 3:**
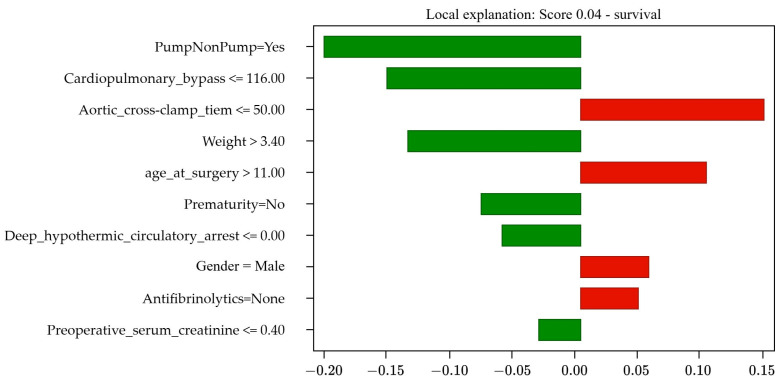
Results of Case Study 2: Correct survival prediction (96%).

The model correctly predicted patient survival with a high probability (96%), as shown in Figure 3. Furthermore, Table 8 shows the factors that positively influenced survival: the use of cardiac pumping with a 19% influence, a cardiopulmonary bypass time of 67 min (15%), adequate weight (13%), the absence of a history of prematurity (7%), and a deep hypothermic circulatory arrest time of 0.0 min (6%). However, the factors that negatively influenced survival included aortic cross-clamp time of 35.0 min (14%), patient age (11%), male sex (6%), and an absence of antifibrinolytics during surgery (5%).

A predominance of positive factors (green bars) over negative ones (red bars) is observed, which explains the high probability of survival.

#### 4.3.3. Incorrect Mortality Prediction

This real case concerns a 23-day-old pediatric patient diagnosed with congenital heart malformation, male, weighing 2.57 kg, not premature, who underwent cardiopulmonary bypass surgery lasting 172.0 min, using aprotinin as an antifibrinolytic drug, with cardiac pumping, no hypothermic circulatory arrest time (0.0 min), and an aortic cross-clamp time of 110.0 min. Preoperative serum creatinine was 0.7. Despite these conditions, the patient survived the operation.

**Figure 4:**
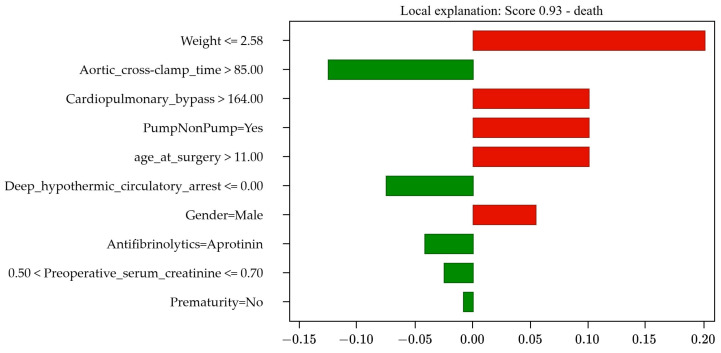
Results of Case Study 3: Incorrect mortality prediction (93%).

However, the model incorrectly predicted mortality with 93% probability, as shown in Figure 4. Furthermore, Table 9 reveals that the factors that erroneously suggested an elevated risk of mortality included a low weight of 2.57 kg (21%), prolonged cardiopulmonary bypass time (9%), age (9%), the use of cardiac pumping (9%), and male sex (6%). On the other hand, the factors that contributed to patient survival were an aortic cross-clamp time of 110.0 min (13%), the absence of hypothermic circulatory arrest time (9%), the use of aprotinin (4%), a preoperative serum creatinine of 0.7 (3%), and the absence of prematurity (1%).

There are two possible explanations for this incorrect prognostic result: (1) the probability of survival, calculated by the system, has a margin of error of 7%, which is higher than the error of 4.22% observed in the GBM model, as detailed in Table 6, and (2) the number of factors influencing survival is equal to the number of factors influencing mortality, and the levels of influence are significantly high in both cases.

### 4.4. Simulation

Here, we present the simulation performed for an actual case of a patient whose initial prognosis indicated a 100% mortality rate and who, unfortunately, died without the simulation scenario of our proposed method.

**Figure 5:**
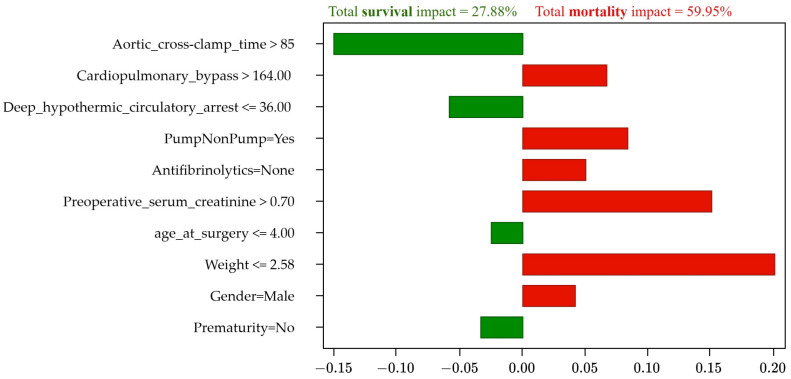
Mortality prediction of the patient with actual (non-simulated) risk factors.

The risk factors for this patient are found in the initial scenario in Table 10. These factors show some unfavorable conditions, highlighting the patient’s low weight of less than 2.58 kg with a 20% influence and an elevated preoperative serum creatinine of more than 0.7 (14.7%). Other influential factors are cardiac pumping (8.7%) and a prolonged cardiopulmonary bypass time (7.2%), presented in the initial scenario in Table 11 and visualized in Figure 5.

Given the significant negative influence of factors such as low weight and high serum creatinine, the values of some factors were modified as part of the simulation process to reduce their impact. The changes made are reflected in the simulated scenario in Table 10. It should be noted that modifying the factor values alters the degree of influence of the factors and, consequently, the outcome of the forecast.

The changes made to the simulation were as follows:

- *Age*: To try to reduce the operative risk, the surgery could be delayed until 12 months of age;
- *Weight*: Increase from 2.4 kg to 2.6 kg, which is feasible by improving the patient’s nutrition to increase their weight;
- *Cardiopulmonary Bypass Time*: Decreasing from 173 min to 130 min is very feasible;
- *Use of Aprotinin*: Aprotinin can be administered to improve intraoperative management and reduce complications;
- *Aortic Clamping Time*: It is possible to reduce its value from 106 min to 80 min;
- *Serum Creatinine*: More effective monitoring of renal function can reduce its value from 0.8 to 0.4.

**Figure 6:**
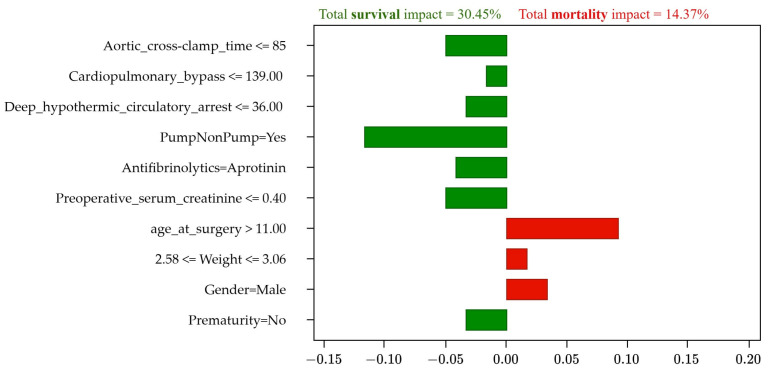
Simulated results of survival (99%).

These changes affected the degree of influence of the risk factors on the prognosis of the simulated case, as detailed in Table 11. Consequently, these changes transformed the patient’s prognosis from 100% mortality (initial scenario) to 99% survival (simulated scenario) as illustrated in Figure 6.

Finally, this simulated scenario shows us that the method proposed in this study could be used to change a patient’s initial unfavorable prognosis through modifications in the risk factors that are feasible to carry out in real life. This would achieve an improved prognosis, and the surgical intervention could be performed with the greatest possible certainty of survival.

## 5. Discussions

### 5.1. Method

This work proposes a 5-step method (data collection, risk factor, prediction, evaluation, explanation, and simulation) to maximize the probability of success of patients undergoing PCHS. The proposed method uses ML algorithms to predict the mortality outcome of a patient in PCHS and ETs to determine the level of influence of the risk factors on the outcome; with this information, the medical specialist can create simulated scenarios by carefully and appropriately changing the values of the risk factors in a way that allows one to find a scenario that provides a high probability of survival for a PCHS case. The proposed method presents a novel approach, combining ML models, ETs, and scenario simulation. In addition, its implementation constitutes an essential tool to manage PCHS cases where uncertainty and a high risk to life demand well-founded information for medical decision-making.

### 5.2. Mortality Prognostic

Numerical evaluations were performed to determine the effectiveness of four ML models (GBM, RF, DT, and NB) in predicting mortality and survival outcomes in cases of PCHS. A dataset of 950 records was used, of which 855 were used for training and 95 (51 surviving patients and 44 deceased) for validation. The GBM and RF models showed the best performances, reaching accuracies of 96% and 95%, respectively, with a sensitivity of 100% and a specificity of at least 90%. These results surpass those obtained by traditional methods such as EuroScore II and others reported in the literature [39].

The lower accuracy in the NB model could be attributed to the dependence between some risk factors, such as cardiopulmonary bypass time and aortic clamping time, which are functionally interconnected. Although the DT model manages to overcome this limitation and presents better results than NB, the RF and GBM models still stand out with an increase of more than 10% in accuracy and 22% in sensitivity compared to DT. This would be explained because these models are more advanced, incorporate techniques that minimize variance and overfitting, and effectively handle diverse data types and complex relationships between them.

### 5.3. Explainability

To address the dichotomy of prediction accuracy versus explainability, independent interpretability techniques are now being used to improve transparency and facilitate an understanding of how a given prognosis was reached. In this study, the LIME algorithm was used to demonstrate that it is possible to make results transparent and to strengthen confidence in making medical decisions. In addition, this technique offers locally valid explanations; that is, it is appropriate for the particular case of each PCHS patient. Therefore, the applicability of LIME was examined in three different prognostic scenarios through case studies:

- Correct mortality prediction: The explanation was overwhelmingly convincing. It highlighted seven negative factors that combined to influence 65% (in favor of mortality), compared to three positive factors that barely reached 10%;
- Correct survival prediction: The explanation was also overwhelmingly convincing, with six positive factors accounting for 62% of the total influence (in favor of survival), while the four negative factors accounted for only 25%;
- Incorrect mortality prediction: This scenario is unlikely, as the forecast model has a high level of reliability (96% accuracy). However, in this case, the interpretation was correct and consistent with the result, with positive (in favor of survival) and negative (in favor of mortality) influences being 28% and 54%, respectively, and the number of positive and negative factors being equal.

These case studies demonstrate that the explainability is consistent across the three scenarios. However, the likelihood ratio of the outcomes in the cases with correct predictions exceeds 1. In the first case, the ratio is 5.5, calculated as (65 − 10)/10; in the second case, 1.5, calculated as (62 − 25)/25; and, in the third case, where the prediction was incorrect, the likelihood ratio is 0.9, which does not exceed 1. This result, combined with a balance between the number of positive and negative factors, could indicate the inaccuracy of the forecast.

### 5.4. Changing the Future and Maximizing Survival

Understanding the influence of risk factors on PCHS outcomes for large groups of pediatric patients requiring congenital cardiac surgery allows researchers to formulate policies and preventive measures to reduce mortality. However, these findings generally apply to the “average patient in the group” and not necessarily to each individual. In this context, the explainability provided by the LIME technique offers a valuable alternative that allows the determination of the specific influence of each factor for each patient individually. With this detailed information, it is possible to take concrete actions to modify the effects of adverse risk factors and thus transform the mortality prognosis into survival. This study has shown that this would be possible thanks to the proposed method, which includes the simulation of patient health scenarios.

To test the method, a simulation of an actual case of mortality with an initial prognosis of 100% (0% survival) was performed, with six negative factors (affecting mortality) and four positive factors (affecting survival), and with a sum of the influence of 59.95% and 27.88% on mortality and survival, respectively. The main negative factors identified were weight and serum creatinine, with influences of 20% and 14.7%, respectively. After a detailed analysis of the patient’s condition, a simulation was performed, adjusting the values of these risk factors, resulting in a simulated scenario where the prognosis was survival with 99% probability, with seven positive and three negative factors that represent 30.45% and 14.37% influence respectively, and with a probability ratio (relative influence) for the survival of 1.1 (greater than 1).

This case study demonstrates that the proposed method is essential for decision-making in PCHS cases. Its use would be important for two reasons: first, it has the potential to reverse a mortality prognosis to survival, and, second, it significantly reduces the uncertainty and risk associated with medical decision-making.

### 5.5. Limitations and Future Work

The results’ accuracy is limited by the data used, the cases considered, the prognostic and explainability models, and the simulation scenarios used. Therefore, the practical application of this method requires the selection of the most appropriate model for the available data and having a high level of knowledge and experience of the PCHS specialist to create feasible scenarios that modify the mortality to survival prognosis. Consequently, a line of future work involves exploring the application of various explainability models in hospitals and clinics to evaluate their impact and accuracy.

Even though the data used in this study (collected from 2003 to 2008) and their outcomes are valid within this research context, new experiments (ML model training, ET evaluation, and simulated scenarios) with the most recent data and additional preoperative and intraoperative factors should be performed to validate their accuracy before applying them in scenarios outside this context.

On the other hand, the probability ratio (relative influence) of the risk factors (positive and negative) associated with the prediction with a value greater than 1 has shown clues about its veracity. This implies future work with more experiments that confirm this finding, even with other available explainability models.

## 6. Conclusions

This study presents an innovative method, based on ML models, ETs, and the simulation of scenarios, to transform the mortality prediction into survival for patients undergoing PCHS. In addition, it has been shown that clinical interventions with a high probability of survival can be achieved through the simulation of feasible scenarios, where risk factors are modified in a personalized way. Additionally, incorporating explanatory models allows physicians to understand and trust the decisions suggested by high-precision prediction models, providing a solid basis when suggesting treatments more tailored to each particular case of pediatric patients.

Finally, the experimental results confirm that this new approach is essential for medical decision-making in patients undergoing PCHS due to its potential use in reversing mortality prognoses, significantly reducing uncertainty and risk, and maximizing favorable clinical outcomes for each patient.

## Figures and Tables

**Table 1:** Prognostic studies of mortality in cardiac surgeries.

Study	Dataset	ML Method	Metric ^1^
[39]	2308 patients from the Children’s Hospital of Zhejiang University School of Medicine.	XGBoost	81%
[38]	920 newborn patients with single ventricle congenital malformations undergoing Norwood surgical procedures (open heart surgery) obtained from the Pediatric Heart Network.	DL GBM RF DT RL	89% 84% 75% 65% 61%
[7]	6520 patients undergoing cardiac surgery with cardiopulmonary bypass obtained from a cardiac surgery center at University Hospital.	Ensemble RF GBM NB SVM	80% ^2^ 79% ^2^ 78% ^2^ 75% ^2^ 74% ^2^
[40]	1482 patients registered at Acıbadem Maslak Hospital.	C4.5 NB KNN	99% 98% 97%
[41]	9269 patients with cardiovascular diseases undergoing cardiothoracic surgery obtained from the German Heart Center Berlin	RNN	88%
[36]	235,000 patients and 295,000 operations provided by the congenital database of the European Association of Congenital Heart Surgeons.	OCTs	86% ^2^
[37]	24,685 pediatric patients aged < 18 years are undergoing coronary heart surgery, including 595 (2.4%) non-survivors from the Shanghai Children’s Medical Center.	XGBoost	88% ^2^

^1^ Accuracy, ^2^ AUC.

**Table 2:** Dataset characteristics.

Attribute	Original Dataset	Preprocessed Dataset	Training and Validation	Test
Number of records	565	950	855	95
Number of risk factors	85	10	10	10
Data types	Numerical and Categorical	Numerical	Numerical	Numerical
Surviving patients	488	488	437	51
Dead patients	77	462	418	44

**Table 3:** Optimal hyperparameters used by the ML models.

GBM	RF	DT	BN
n_estimators = 157 max_depth = 53 min_samples_split = 5 min_samples_leaf = 9	n_estimators = 285 max_features = log2 max_depth = 11 min_samples_split = 6 min_samples_leaf = 2	Criterion = Entropia max_depth = 39 min_samples_split = 11 min_samples_leaf = 16	Alpha = 0.00005

**Table 4:** Metrics used for evaluating the ML models.

Metric	Description	Formula
Accuracy	Percentage of patients with correct predictions.	$\frac{TP+TN}{TP+FP+FN+TN}$
Sensitivity	Percentage of patients who died and obtained a mortality prediction.	$\frac{TP}{TP+FN}$
Specificity	Percentage of patients who survived and obtained a survival prediction.	$\frac{TP}{TN+FP}$

Note: *TP* (True Positive) is the number of cases of patients with mortality that the model correctly predicted; *TN* (True Negative) is the number of cases of patients who survived and were correctly predicted by the model; *FP* (False Positive) is the number of patients who survived and were predicted by the model as mortality; *FN* (False Negative) is the number of patients with mortality and who were predicted by the model as survival.

**Table 5:** Confusion matrix for the ML models.

Class	GBM		RF		DT		BN	
	(1)	(0)	(1)	(0)	(1)	(0)	(1)	(0)
Mortality (1)	44	0	44	0	34	10	24	20
Survival (0)	4	47	5	46	6	45	15	36

**Table 6:** Performance of ML models in predicting the outcome of PCHS.

Metrics	GBM	RF	DT	BN
Accuracy	**95.79%**	94.74%	83.16%	63.16%
Sensitivity	**100.00%**	**100.00%**	77.27%	54.55%
Specificity	**92.16%**	90.20%	88.24%	70.59%

**Table 7:** Influence of risk factors observed in Case Study 1.

Decision Rule	Influence Level
Weight <= 2.58	−21%
Preoperative_serum_creatinine > 0.70	−13%
PumpNonPump = Yes	−9%
Prematurity = Yes	−8%
139.00 < Cardiopulmonary_bypass <= 164.00	−7%
50.00 < Aortic_cross-clamp_tiem <= 68.00	−4%
13.00 < Deep_hypothermic_circulatory_arrest <= 36.00	+4%
Gender = Male	−3%
4.00 < age_at_surgery <= 6.00	+3%
Antifibrinolytics = Aprotinin	+3%

**Table 8:** Influence of risk factors observed in Case Study 2.

Decision Rule	Influence Level
PumpNonPump = Yes	+19%
Cardiopulmonary_bypass <= 116.00	+15%
Aortic_cross-clamp_tiem <= 50.00	−14%
Weight > 3.40	+13%
age_at_surgery > 11.00	−11%
Prematurity = No	+7%
Deep_hypothermic_circulatory_arrest <= 0.00	+6%
Gender = Male	−6%
Antifibrinolytics = None	−5%
Preoperative_serum_creatinine <= 0.40	+3%

**Table 9:** Influence of risk factors observed in Case Study 3.

Decision Rule	Influence Level
Weight <= 2.58	−21%
Aortic_cross-clamp_time > 85.00	+13%
Cardiopulmonary_bypass > 164.00	−9%
PumpNonPump = Yes	−9%
age_at_surgery > 11.00	−9%
Deep_hypothermic_circulatory_arrest <= 0.00	+7%
Gender = Male	−6%
Antifibrinolytics = Aprotinin	+4%
0.50 < Preoperative_serum_creatinine <= 0.70	+3%
Prematurity = No	+1%

**Table 10:** Risk factors for a pediatric patient (actual vs. simulated).

Risk Factor	Actual	Simulated
Age at surgery	3	12
Gender	0	0
Weigth	2.4	2.6
Prema-turity	0	0
CPB min	173	130
Antifinrinolytics	0	2
Pump-NonPump	1	1
DHCA-min	32	32
Aox_time	106	80
Pre_oCr	0.8	0.4

**Table 11:** Influence of the risk factors of a pediatric patient (actual vs. simulated).

Scenario	Actual	Simulated
Aortic_cross-clamp_time	14.6%	4.4%
Cardiopulmonary_bypass	−7.2%	1.4%
Deep_hypothermic_circulatory_arrest	6.5%	3.3%
PumpNonPump	−8.7%	11.6%
Antifibrinolytics	−5.2%	3.7%
Preoperative_serum_creatinine	−14.7%	4.4%
age_at_surgery	3.2%	−8.8%
Weight	−20.0%	−2.1%
Gender	−4.1%	−3.5%
Prematurity	3.6%	1.6%
**Prediction result**	**Mortality 100%**	**Survival 99%**

## Data Availability

The dataset used in this article is publicly available, and its source is indicated in the article.
